# Redefining flow measurement in centrifugal pump: the case for RPM and power consumption-based estimation

**DOI:** 10.1051/ject/2025038

**Published:** 2025-12-17

**Authors:** Ignazio Condello

**Affiliations:** 1 University of Insubria Via Ravasi, 2 21100 Varese VA Italy

**Keywords:** Mechanical circulatory support, Centrifugal pumps, Flow estimation, RPM, Power consumption

## Abstract

Mechanical circulatory support (MCS) devices, particularly those utilizing centrifugal pumps (CP), are crucial in sustaining patients with severe cardiac conditions. Traditional flowmeters, integral to these systems, present considerable challenges, including substantial physical bulk, complex wiring, and a dependency on robust backup systems. These challenges become acute in portable applications, where compact, reliable, and discrete solutions are essential for enhancing patient mobility and quality of life. This paper proposes a strategic shift towards integrating RPM (Revolutions Per Minute) and power consumption-based flow estimation as a standard in MCS device design. This approach reduces device complexity, enhances reliability, and diminishes the need for invasive hardware, aligning with broader goals of patient safety and device efficiency. By adopting RPM and power consumption-based estimation, MCS devices can achieve improved ergonomics, fail-safe operational integrity, and enhanced energy efficiency, which are crucial for achieving patient-centric outcomes. This advancement signals a move towards more intelligent, adaptive medical devices that could redefine standards in mechanical circulatory support.

*Dear Editor*,

As we navigate the complex landscape of mechanical circulatory support (MCS) devices with the centrifugal pump (CP) use, it becomes increasingly clear that traditional flowmeters, though fundamental to these systems, present significant challenges and limitations. The physical bulk of these instruments, the intricate web of necessary wiring, and their vulnerability requiring robust backup systems, all posing substantial constraints. These limitations are particularly pronounced in portable applications, where the need for compact, reliable, and unobtrusive equipment is paramount to patient mobility and quality of life. In response to these critical issues, there emerges a compelling case for rethinking traditional approaches [1]. While celebrating these advancements, we consider a strategic redirection towards integrating rate per minute (RPM) and power consumption-based flow estimation as a standard for CP in MCS device design. This approach not only complements the existing technological landscape but also aligns with the broader goal of enhancing patient safety and device efficiency. The technique of estimating blood flow from RPM and power consumption is rooted in a well-defined engineering principle. It utilizes a modified Bernoulli equation where the flow rate ***Q*** is estimated by:

$$Q=k\;\times \sqrt{P\times N}.$$

Here, ***P*** represents power consumption and ***N*** the rotational speed, with ***k*** being a device-specific coefficient that encapsulates the pump characteristics and fluid dynamics. This formula underscores an elegant engineering solution to a clinical challenge, offering a streamlined, less invasive measurement technique that inherently enhances device reliability and patient safety [2].

## Perspective on clinical benefits

1. *Ergonomic design and mobility*: The simplification in device architecture by removing traditional flowmeters significantly reduces weight and enhances the ergonomic design of portable MCS devices such as VADs. This directly correlates to improved patient mobility and comfort, key factors that impact the overall quality of life and clinical outcomes.
2. *Reliability and safety*: By providing a fail-safe mechanism through RPM and power-based estimations, these devices can maintain operational integrity even in the event of primary sensor failure. This built-in redundancy is crucial in life-support systems, where continuous performance is non-negotiable.
3. *Sustainability and operational efficiency*: Reducing the mechanical complexity of MCS devices not only lowers the risk of mechanical failures but also decreases the energy demands of the system. This is particularly advantageous in enhancing the longevity and operational times of battery-powered units, thereby supporting longer and safer patient use outside clinical settings.

The adoption of RPM and power consumption-based flow estimation in MCS devices, especially in VADs, illustrates a move towards more intelligent, adaptive medical devices. Standardizing this approach could catalyze further innovations and lead to universally safer and more efficient MCS solutions [3]. Therefore, I advocate for increased research focus and regulatory support for technologies that integrate these engineering principles, which are poised to redefine the landscape of mechanical circulatory support. As we continue to advance in our technological capabilities, it is imperative that we also reframe our perspectives to prioritize solutions that enhance both the clinical efficacy and the daily living conditions of patients. RPM and power consumption-based flow estimation exemplifies such a solution, embodying the fusion of engineering innovation with clinical benefit. Let us embrace this opportunity to set new standards in the design and functionality of MCS devices, aiming for a future where technology and patient care evolve in tandem.
